# *Plectranthus*‐derived Abietanes as Protein Kinase C‐δ Activators: In Silico Design, Human Serum Albumin Interaction, and Stability Evaluation

**DOI:** 10.1002/cbdv.202500670

**Published:** 2025-10-08

**Authors:** Vera M. S. Isca, Milan Nikolić, Nenad Filipović, Mattia Mori, Patricia Rijo

**Affiliations:** ^1^ CBIOS – Universidade Lusófona’s Research Center for Biosciences & Health Technologies Universidade Lusófona Lisboa Portugal; ^2^ Faculty of Chemistry University of Belgrade Belgrade Serbia; ^3^ Faculty of Agriculture University of Belgrade Belgrade Serbia; ^4^ Department of Biotechnology, Chemistry and Pharmacy Università Degli Studi di Siena Siena Italy; ^5^ Research Institute for Medicines (iMed.ULisboa), Faculty of Pharmacy Universidade De Lisboa Lisboa Portugal

**Keywords:** abietane diterpene, antioxidant activity, HSA, molecular docking, PKC‐δ activation

## Abstract

Cancer remains a major global health challenge. Among protein kinases (PKCs), PKC‐δ acts as a tumor suppressor in colon cancer and represents a valuable therapeutic target. Human serum albumin (HSA) is gaining attention as an efficient drug carrier, while *Plectranthus* spp. offers a rich source of bioactive compounds. One such molecule is 7α‐acetoxy‐6β‐hydroxyroyleanone (Roy, **1**), a cytotoxic abietane diterpenoid with modifiable hydroxyl groups, making it a promising scaffold for drug development. This study aimed to design a theoretical library of Roy **1** derivatives targeting PKC‐δ. Hydroxyl groups at positions C6 and C12 were modified to explore interactions through molecular docking against the PKC‐δ regulatory domain (1PTR). Compound **16** emerged as the most promising candidate. Additionally, the binding of Roy **1** to HSA was evaluated by steady‐state fluorimetry, revealing moderate affinity near Trp‐214 and enhancing the thermal stability of the complex. Roy **1** exhibits excellent aqueous stability (0.1 mM, pH 7.4, 37°C), with similar results for two benzoylated derivatives (RoyBz and Roy12Bz), and no ester hydrolysis was detected. These findings highlight Roy **1**’s potential as a stable, bioactive lead compound for developing PKC‐δ‐targeted therapeutics, with HSA as a suitable delivery vehicle.

## Graphical abstract

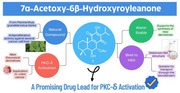

## 1 Introduction

Cancer stands as one of the most predominant causes of death worldwide [1]. The protein kinase (PKC) family plays a pivotal role in tumorigenesis and metastatic dissemination [2]. Clinical trials involving PKC modulators have generally demonstrated limited success, likely due to the complex and diverse biological functions of PKC isozymes. These functions can be both redundant and opposing, with expression levels varying significantly across different cancer types and isoforms displaying a high degree of structural similarity [3]. PKC‐δ has been widely characterized as a pro‐apoptotic and anti‐proliferative kinase [4, 5]. However, PKC‐δ promotes therapy resistance in several tumor models, including lung, pancreatic, and liver cancers [2].

Human serum albumin (HSA) has emerged as a versatile and invaluable drug carrier with significant therapeutic potential. It is a soluble and monomeric, multi‐domain macromolecule abundantly present in human blood plasma, where it plays a key role in pH buffering and maintaining oncotic pressure [6]. HSA functions as the primary transporter of a wide range of endogenous and exogenous compounds. Its structural features, including hydrophobic binding pockets, a free thiol group, and surface‐exposed termini, make it particularly well‐suited for the targeted delivery of diverse therapeutic agents. HSA has shown promise as a carrier for drugs used in the diagnosis and treatment of various conditions, including carcinomas, hyperglycemia, infectious diseases, and rheumatoid arthritis. HSA can facilitate drug accumulation in tumors or inflamed tissues through receptor‐mediated transport mechanisms. Furthermore, HSA exhibits notable antioxidant properties that can be leveraged to improve therapeutic efficacy and reduce drug‐induced toxicity [7, 8]. In addition, HSA plays an essential role in enhancing the circulation and bioavailability of anticancer drugs within the bloodstream [9].

**FIGURE 1:**
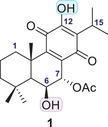
Chemical structure of the abietane diterpenoid 7α‐acetoxy‐6β‐hydroxyroyleanone (Roy, **1**), isolated from *Plectranthus grandidentatus*. The hydroxyl groups at positions C6 and C12, selected for structural modification, are highlighted.

Plants represent a valuable source of novel biologically active compounds with significant potential for promoting human health and treating various diseases [10]. The genus *Plectranthus* L'Hér., commonly referred to as spurflowers, belongs to the Lamiaceae family and comprises more than 300 species, widely distributed across the Old World tropics and subtropical regions [11]. Many *Plectranthus* species have been traditionally used in folk medicine to manage a range of conditions, including neurological and blood complaints, infections and fevers, pain, and inflammation [12]. *Plectranthus* is particularly rich in bioactive compounds, such as abietane‐type terpenoids. Abietanes have demonstrated a broad spectrum of biological activities, including antibacterial, antitumoral, anti‐inflammatory, antidiabetic, and antioxidant effects. Structurally, Abietane diterpenoids are characterized by a tricyclic abietane carbon skeleton substituted with various functional groups [13]. The abietane diterpenoid 7*α*‐acetoxy‐6*β*‐hydroxyroyleanone (Roy, **1,** Figure 1) isolated from *P. grandidentatus* Gürke has exhibited notable anti‐proliferative activity against several cancer cell lines [14, 15].

The cytotoxic potential of bioactive compounds can be effectively assessed using in silico approaches. Due to its speed and cost‐effectiveness, molecular docking serves as a valuable alternative for high‐throughput screening of potential drug candidates [16]. In the present study, molecular docking and molecular dynamics (MD) were employed to investigate the impact of structural modifications on the hydroxyl groups of royleanone **1**, with a focus on its ability to activate PKC‐δ. A recent in silico analysis highlighted the potential of Roy **1** to interact with PKC isoforms, suggesting that subtle modifications in its substitution pattern could enhance isoform‐specific binding, particularly toward PKC‐δ [17]. In light of earlier findings, the derivative 7*α*‐acetoxy‐6*β*‐benzoyloxy‐12‐*O*‐benzoylroyleanone (RoyBz) has emerged as the first selective PKC‐δ activator. RoyBz binds specifically to the PKCδ‐C1‐domain and has been shown to inhibit the proliferation of colon cancer cells by inducing apoptosis [18]. Additionally, RoyBz shows the ability to inhibit both mitochondrial respiration and glycolysis, thereby reinforcing its potential as an antitumor agent in colon cancer therapy [19]. Extending this approach, the present work aims to investigate novel royleanone‐based derivatives capable of modulating PKC‐δ for the treatment of colon cancer. Additionally, the interaction between Roy **1** and HSA was evaluated in vitro, alongside an assessment of its aqueous stability, to support future pharmaceutical formulation strategies.

## 2 Results and Discussion

### 2.1 Molecular Docking on PKCδ‐C1‐domains

In this study, molecular docking was employed as a key tool to support the discovery of novel royleanone derivatives with potential therapeutic application in colon cancer, specifically through PKC‐δ modulation. Knowledge of the ligand‐binding site prior to docking significantly enhances the efficiency and accuracy of the docking process [20]. In this case, the binding site was well‐characterized, as the selected crystallographic structure of the PKC‐δ‐C1 domain (PDB ID:1PTR) from the protein data bank [21] was co‐crystallized with phorbol‐13‐acetate (PA), an endogenous activator of PKC. An initial comparative docking study was conducted using three widely used docking programs: FRED, GOLD, and AutoDock. The accuracy and reliability of each program were assessed by comparing the predicted ligand‐binding poses to two benchmarks: the crystallographic pose of PA [21] and the reported docking results of the known PKC‐δ activator RoyBzBz [18].

**FIGURE 2:**
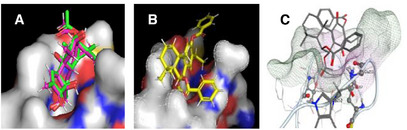
(A) Pose of the natural inhibitor phorbol‐13‐acetate (PA) (green) complexed in protein kinase (PKC)‐δ (1PTR.pdb crystallographic structure) and of the PA design in VIDA software (pink); (B) Best pose obtained for RoyBz and (C) previously published data for RoyBz [19].

Among the evaluated tools, FRED software [22] demonstrated superior performance by accurately reproducing the binding pose of the crystallized PA molecule (Figure 2A), as well as the previously reported docking configuration of RoyBz [18] (Figure 2B,C). While GOLD and AutoDock are both well‐established molecular docking tools, they were not selected due to their lower accuracy in reproducing the crystallographic pose of PA. GOLD showed deviations in ligand orientation, while AutoDock produced inconsistent results across runs. In contrast, FRED consistently aligned with experimental data. As a result, FRED was selected as the primary docking platform for the subsequent screening studies.

Libraries of theoretical compounds were constructed for docking screening with the dual objectives of increasing structural diversity and exploring the reactive hydroxyl groups of compound **1** (highlighted in Figure 1) for potential esterification. Nineteen substituents (**S1**–**S19**, Table 1) were selected to generate novel esters, drawing inspiration from literature‐reported bioactive moieties. The selection of ester types followed a rational design strategy aimed at covering a broad range of physicochemical and steric properties, including lipophilicity, electronic effects (e.g., π‐interactions), flexibility, and steric hindrance. This diverse set of substituents (Table 1) was intended to identify favorable interactions within the PKC‐δ binding site and guide the design of derivatives with enhanced specificity and activity.

**TABLE 1:** Summary of ester substituents (**S1**–**S19**) used for Roy **1** derivatization.

Code	Substituent type	Representative group	Design rationale	Code	Substituent type	Representative group	Design rationale
**S1**	Simple acyl	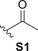	Baseline lipophilicity and minimal steric hindrance	**S13‐S14**	Spacer group	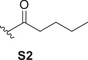 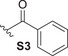	Conformational flexibility and extended reach
**S2**	Aliphatic chain	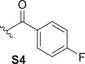	Flexibility and increased chain length	**S15**	Bulky aliphatic	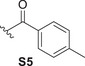	High steric hindrance and hydrophobicity
**S3**	Aromatic benzoyl	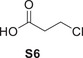	Enhanced rigidity and π‐stacking potential and aromaticity	**S16**	Bulky aromatic	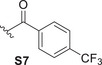	π‐system extension and rigidity
**S4‐S5**	Electron‐donating	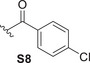 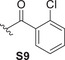	Increased electron density and π‐donor properties	**S17‐S18**	Heterocyclic	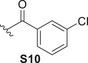 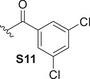	π‐interactions, polar interactions, and hydrogen bonding
**S6**	Electron‐withdrawing	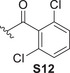	Flexibility, strong lipophilicity, and electron deficiency	**S19**	Hetero‐aromatic	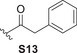	Rigid, planar moiety and hydrogen bonding
**S7‐S12**	Electron‐withdrawing	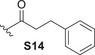 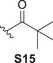 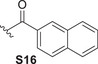 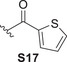 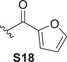 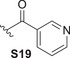					Strongly lipophilic and polar and π‐acceptor properties

**FIGURE 3:**
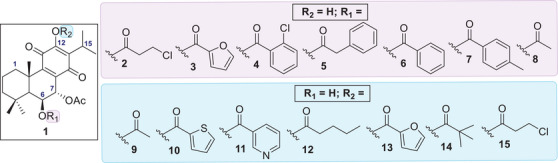
Top hits derivatives revealed from the docking screening: C6 derivatives 2–8 (in purple) and C12 derivatives 9–15 (in blue).

Molecular docking screening revealed several promising candidates with higher predicted binding affinities than the known selective PKC‐δ activator RoyBz. When both hydroxyl positions were derivatized simultaneously, only one compound, the previously reported 7*α*,6*β*‐diacetoxy‐12‐*O*‐acetylroyleanone [23], displayed favorable results. This finding suggests that maintaining at least one free hydroxyl group may be important for binding affinity. To systematically assess the impact of structural modifications, each hydroxyl position was modified independently, while the other remained unaltered. As shown in Figure 3, theoretical derivative **2** emerged as the most promising compound. Overall, esterification at the C6‐OH position resulted in better docking scores than modifications at C12, suggesting a higher potential for PKC‐δ activation through derivatization at C6. The C6 position appears to tolerate a wide range of substituents, including linear chains, heterocyclic rings, and substituted or unsubstituted benzene rings (compounds **2** ‐ **8**, highlighted in purple in Figure 3). In contrast, derivatives at C12 showed a preference for aliphatic chains or aromatic rings with heteroatoms (analogs **9**–**15**, highlighted in blue in Figure 3).

**FIGURE 4:**
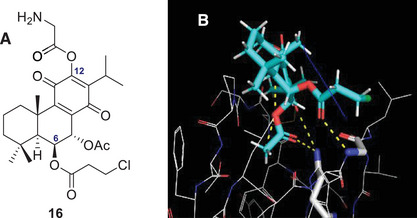
Best hit obtained from protein kinase (PKC)‐δ in silico screening: (A) derivatives 16 and (B) top‐ranked pose and hydrogen interaction between 16 and the amino acid residues from PKC‐δ.

To address a major limitation of this compound, its poor water solubility [24, 25], a new strategy involving amino acids was explored. These organic molecules, which feature both an amino group (‐NH_2_) and a carboxylic acid group (‐COOH), along with distinct side chains, offer varying degrees of hydrophilicity. For instance, amino acids such as lysine and arginine have positively charged side chains, while serine contains a hydroxyl group, all of which contribute to enhanced water solubility [26, 27]. Previous studies have demonstrated that conjugating bioactive molecules with amino acids can improve both their pharmacological properties and solubility [26, 28]. Likewise, the introduction of an amino acid was investigated to improve water solubility. Based on this rationale, a new library of theoretical derivatives was developed using the top‐scoring compound (theoretical derivative **2**) as the template. In this new series, amino acids were introduced at position C12, including proline, glycine, alanine, tryptophan, and aspartate. Among these, the glycine‐containing derivative (compound **16**, Figure 4a) showed the most favorable interactions, forming three hydrogen bonds with PKC‐δ. These involved the acetoxy group at C‐17 and carbonyl at C‐14 interacting with Gln257 and the carbonyl of the propionic side chain (C‐21) forming a bond with Gly253 (Figure 4b).

These three hydrogen bonds are also present in other derivatives, including compound **2**. However, the glycine derivative (compound **16**) forms an additional hydrogen bond through its amine group with Met238, potentially enhancing its binding affinity. Notably, compound **16** appears to exhibit a stronger predicted affinity for PKC‐δ than the other tested derivatives, although its isoform selectivity has not yet been determined. These findings provide important insights that can guide the selection of promising candidates for future synthesis and biological evaluation.

### 2.2 Molecular Dynamics Simulations of Compound **16**

**FIGURE 5:**
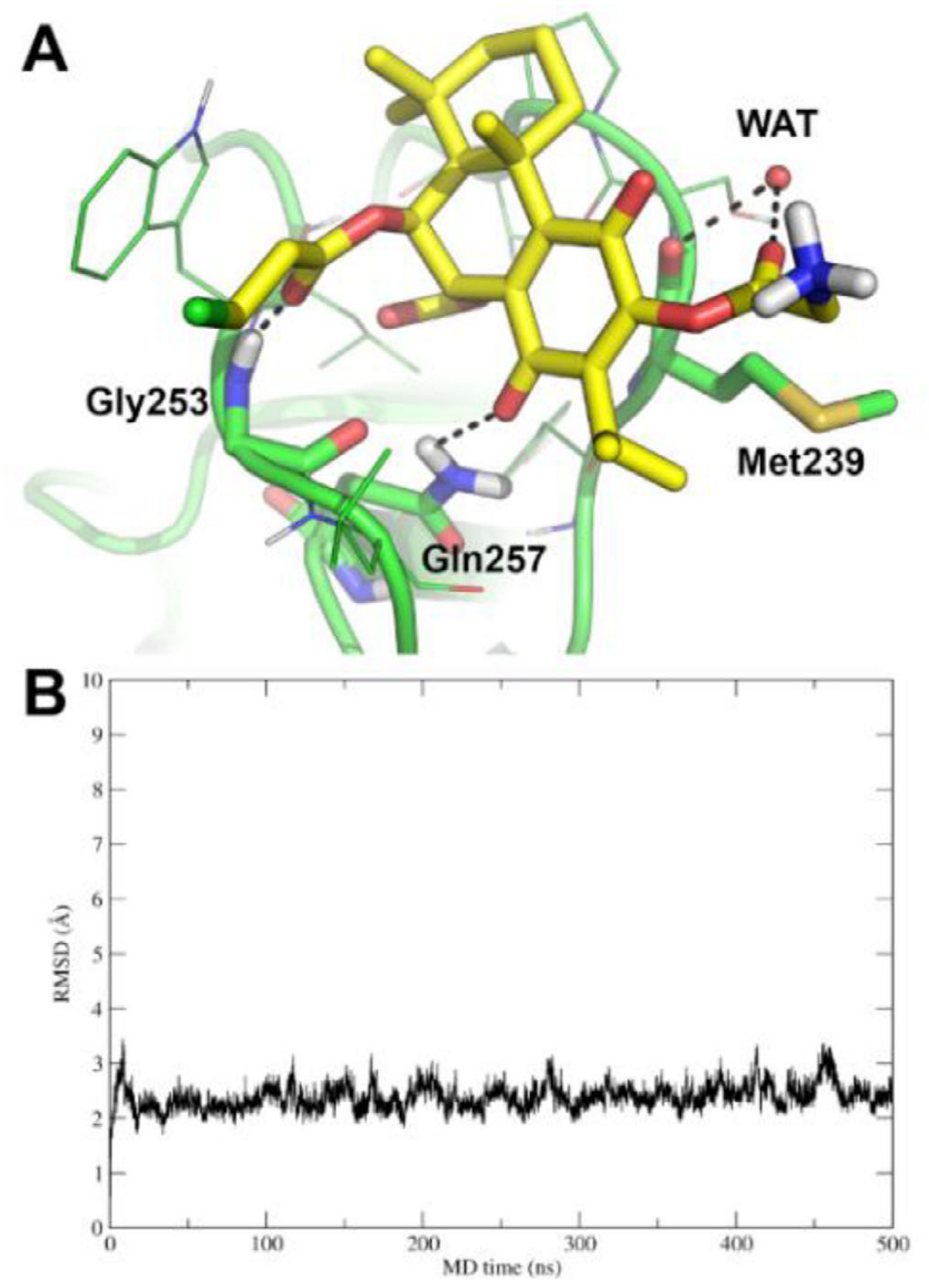
Molecular dynamics (MD) simulations on the docking pose of **16** to protein kinase (PKC)‐δ. (A) Representative frame as extracted from cluster analysis of MD frames. **16** is shown as yellow sticks, PKC‐δ as green cartoon and lines, while residues H‐bonded by **16** are shown as sticks and are labelled. H‐bonds are highlighted by black dashed lines, the bridging water molecule is shown as a small red sphere, and is labelled WAT. Residues within 4 Å from **16** are shown as lines; other residues are hidden for the sake of clarity. (B) Root‐mean square deviation (RMSD) of the PKC‐δ/**16** complex along MD simulation time.

To confirm the docking pose of **16**, 500 ns of unrestrained MD simulations were run in explicit solvent. The MD trajectory was then analysed for the binding mode of **16** through frames cluster analysis and calculation of the root‐mean square deviation (RMSD). Results unequivocally confirm that the binding mode of **16**, such as predicted by molecular docking, is highly stable in the MD simulation. Indeed, the most populated cluster of frames is composed of 94.9% of MD frames, whose representative structure is shown in Figure 5A. Overall, **16** interacts with PKC‐δ with a pose that is highly similar to that of the crystallographic ligand PA [29] by direct H‐bonds to Gly253 and Gln257, as well as a water‐bridged H‐bond to the backbone of Met239. In addition to cluster analysis, the conformational stability of the PKC‐δ/**16** complex was assessed by RMSD calculation (Figure 5B).

### 2.3 Fluorimetric Analysis of the HSA/Roy Interactions

**FIGURE 6:**
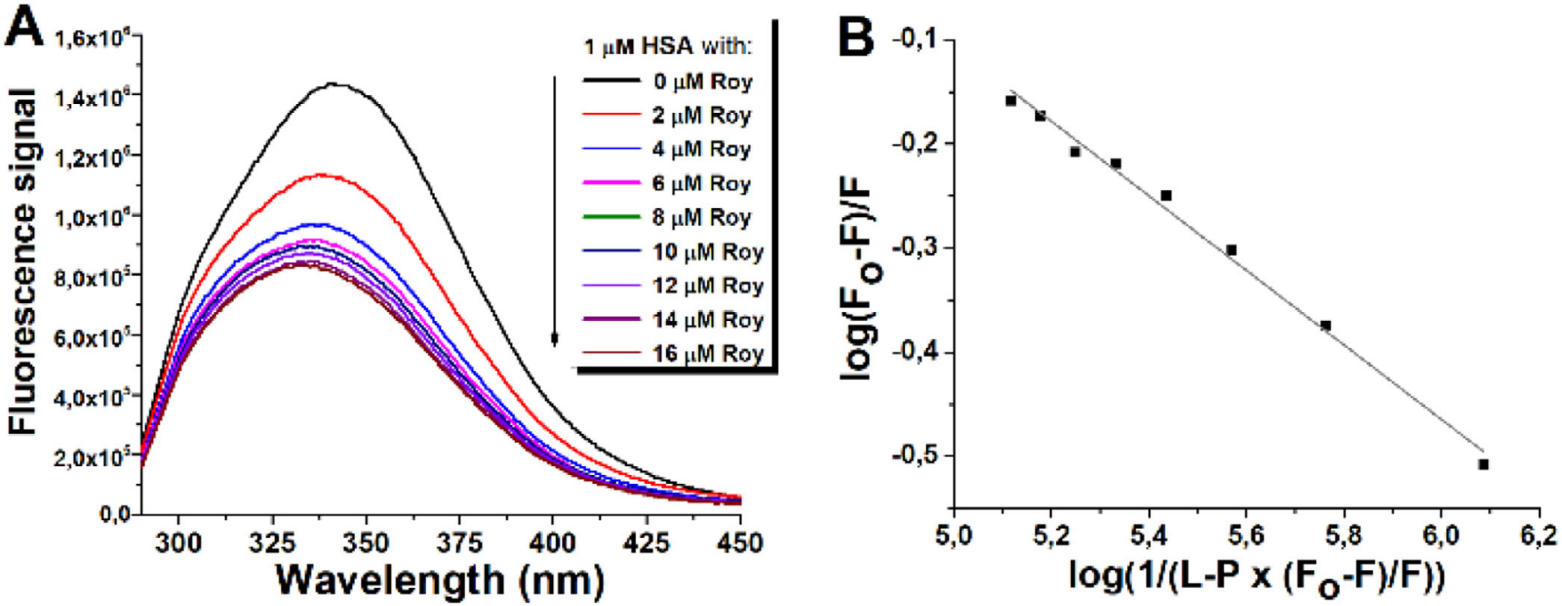
(A) Representative emission spectrum (excitation at 280 nm) of human serum albumin (HSA) (protein) in the absence and the presence of different concentrations of Roy 1 (ligand) at pH 7.2. (B) Plot for determination of the binding constant of the HSA/1 complex at 37°C (mean values from two independent experiments performed in duplicate; standard deviations were less than 5%).

Given the importance of HSA for the delivery of anticancer drugs through the bloodstream [9], interactions between **1** and this protein were studied in vitro under simulated physiological conditions. Ligand binding often induces quenching of intrinsic protein fluorescence. HSA has only one Trp residue (Trp‐214 at subdomain IIA) and 18 Tyr residues [30]. The interactions between HSA and compound **1** caused the concentration‐dependent reduction of the intrinsic fluorescence of protein, followed by a blue shift of the emission maximum from 342 to 333 nm (Figure 6A). Using the quenching data (Figure 6B), a binding constant for the HSA/**1** complex was determined to be 6.5 × 10^4^ M^−1^ at 37°C. The resulting association constant was of moderate strength, similar to most anticancer drugs that have been shown to bind to HSA, mainly to subdomain II [31].

**FIGURE 7:**
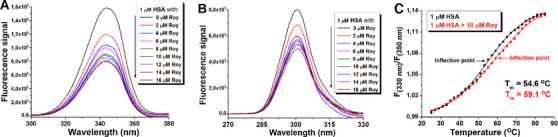
Representative synchronous fluorescence spectra of human serum albumin (HSA) with (A) Δλ of 60 nm (Trp) and with (B) Δλ of 15 nm (Tyr) in the presence of increasing concentrations of Roy 1 at pH 7.2; and (C) determination of the melting points of HSA and the HSA/1 complex at pH 7.2; the ratio of fluorescence intensities at 330 and 350 nm (F330/F350) was calculated (mean values from two independent experiments; standard deviations were less than 5%). The inflection point on the curve corresponds to the protein melting point (T_m_).

Synchronous fluorescence is a valuable tool for separating the contribution of Trp and Tyr residues to total protein intrinsic fluorescence. The fluorescence of HSA with Δλ (Δλ = λem—λex) of 60 and 15 nm is characteristic of Try and Tyr, respectively [32]. From Figures 6A and 6B, it was evident that the binding of compound **1** induced greater Trp residue fluorescence quenching compared to the decrease in fluorescence arising from Tyr residues, with no significant shifts of emission maxima, indicating that bound Roy **1** was situated closer to Trp residues. Therefore, the observed blue shift seen in the HSA fluorescence spectrum (Figure 7A) is due to preferential quenching of the Trp‐214 residues by royleanone **1** binding. In contrast, the relative contribution of Tyr residues to protein fluorescence increases upon ligand binding (Figure 7B).

Experiments comparing HSA and HSA/**1** complex properties have shown a difference in their thermal stability. The calculated melting point was higher (on average 4.5°C or 8.2%) in the complex than in the protein alone (Figure 7C). This result indicated that Roy **1** binding stabilized HSA.

Taken together, these results point to moderate affinity binding of compound **1** to human serum albumin (HSA) at subdomain IIA (Sudlow site I), increasing the protein's thermal stability. This suggests that HSA could be a suitable carrier protein for transporting Roy 1 through systemic circulation.

### 2.4 In Vitro Antioxidant Capacity

The antioxidant capacity of Roy **1** was evaluated alongside antioxidant standards, Vitamin C, and Trolox, using six different assays: 2,2‐diphenyl‐1‐picrylhydrazyl (DPPH), 2,2′‐azino‐bis(3‐ethylbenzothiazoline‐6‐sulfonic acid) (ABTS), nitric oxide (NO), total antioxidant capacity assay (TAOC), oxygen radical absorbance capacity (ORAC), and hydroxyl radical antioxidant capacity (HORAC). DPPH, ABTS, and NO are spectrophotometric assays that measure the ability of compounds to quench DPPH stable radical, in situ formed ABTS cation radical, and ^●^NO radical, respectively. TAOC is a spectrophotometric redox‐based assay that assesses the total antioxidant capacity through metal ion reduction. For consistency, antioxidant activity in these assays is expressed as half‐maximal inhibitory concentration (IC_50_) values for DPPH, ABTS, and NO assays and as EC_50_ values for the TAOC test. HORAC and ORAC are fluorometric assays that evaluate the capacity of compounds to scavenge hydroxyl and peroxyl radicals, respectively, with results expressed as Trolox equivalents (TEs). As shown in Table 2, Roy **1** exhibited low antioxidant capacity (about 7–10 times less than selected standard antioxidants), showing modest activity only in the ^●^NO scavenging assay compared to Vitamin C.

**TABLE 2:** In vitro antioxidant capacity of Roy **1**, Vitamin C, and Trolox.

Parameter (Units)	IC_50_ (mM)			EC_50_ (mM)	(TE)	(TE)
**Test**	**DPPH**	**ABTS**	**NO**	**TAOC**	**ORAC**	**HORAC**
**Roy 1**	>1.5	∼1.3	0.800	>2.0	0.178	0.142
**Vitamin C** ^[a]^	0.275	0.240	0.702	0.234	0.895	0.911
**Trolox** ^[a]^	0.211	0.143	0.160	>1.25	1	1

^[a]^ In DMSO (as for all other samples), numerical data represent means ​​from two independent experiments performed in duplicate (standard deviations were below 7%).

### 2.5 Evaluation of Roy **1** Stability in Aqueous Medium

To support future pharmaceutical formulation studies using Roy **1** as a lead compound, its stability in aqueous media was investigated. Stability testing was conducted in phosphate‐buffered saline (PBS, pH 7.4) at 37°C, a physiologically relevant environment [33, 34]. As previously reported for other royleanones [25, 35], Roy **1** has poor water solubility and thus limited solubility in PBS.[24] To address this, co‐solvents were employed. Acetonitrile (ACN, 17%) [36] and dimethyl sulfoxide (DMSO, 10%) [34, 37] were used to enhance solubility. Stock solutions of Roy **1** were prepared at 0.1 mM in PBS/ACN (17%), 0.4 mM in PBS/ACN (17%), and 0.4 mM in PBS/DMSO (10%). These were incubated at 37°C for periods ranging from 10 to 40 days and monitored over time. The concentration of Roy **1** was determined by high‐performance liquid chromatography‐diode array detector (HPLC‐DAD) at a detection wavelength of 270 nm using a calibration curve. Regression analysis showed good linearity (r^2^ = 0.9960). The retention time (Rt), regression equation, correlation coefficient (r^2^), limit of detection (LOD), and limit of quantification (LOQ) are summarized in Table 3.

**TABLE 3:** Parameters of calibration curve for compound **1**.

Rt (min)	Regression equation	Correlation coefficient (r^2^)	LOD (µg/mL)	LOQ (µg/mL)
5.6	*y* = 25793*x* + 668.36	0.9960	6.6	19.9

**FIGURE 8:**
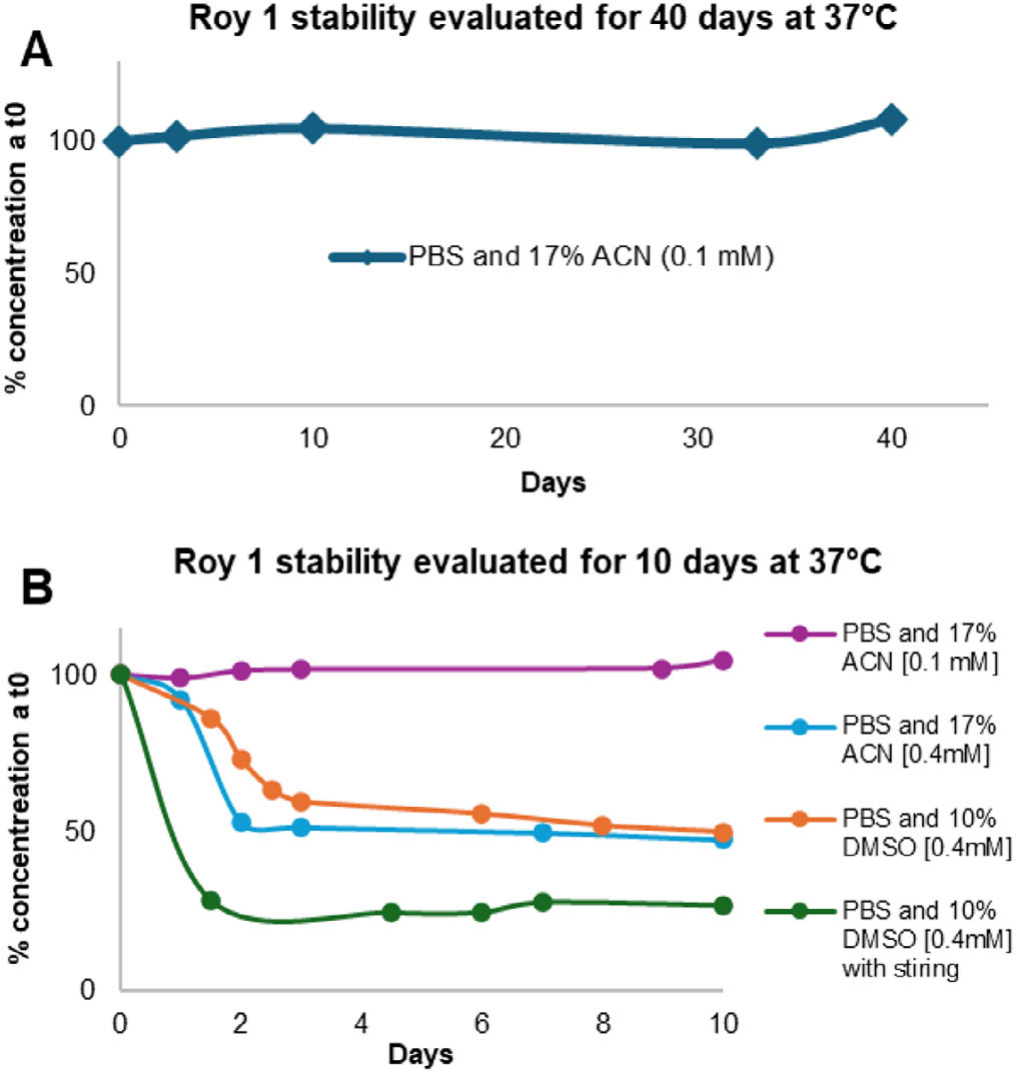
Stability in aqueous media phosphate‐buffered saline (PBS buffer, pH 7.4) of Roy **1** (A) at 0.1 mM and 37°C over a 40‐day period and (B) at 0.1 mM and 0.4 mM in PBS/ACN or PBS/DMSO, and 37°C over a 10‐day period. The initial concentration was quantified by measuring the high‐performance liquid chromatography‐diode array detector (HPLC‐DAD) chromatogram absorbance peak area at 270 nm. Data is shown as % of initial concentration [37].

The results indicate that compound **1** remains fully stable in aqueous medium at a concentration of 0.1 mM, pH 7.4, and 37°C, with no degradation detected over a 40‐day period (Figure 8A). However, at a higher concentration (0.4 mM), a noticeable reduction in the peak corresponding to compound **1** was observed as early as day 2 of the experiment (Figure 8B). This effect occurred in both solvent systems (PBS/ACN and PBS/DMSO) but was more pronounced in PBS/DMSO, particularly under stirring conditions. Importantly, no additional peaks were detected in any chromatograms, suggesting compound **1** underwent precipitation rather than degradation. This precipitation tendency, especially at elevated concentrations, is consistent with the compound's low water solubility. At 0.1 mM in PBS/ACN, approximately 16% of Roy **1** precipitated, with minimal variation over time. In contrast, at 0.4 mM in PBS/ACN, precipitation increased from 35% on day 1% to 66% by day 2. The collected precipitate was reinjected into the HPLC, confirming the exclusive presence of compound **1**. These findings support the conclusion that Roy **1** is chemically stable under the tested conditions and that its apparent concentration loss is due to precipitation, not decomposition, across the tested range (0.1–0.4 mM).

Pires et al. highlighted that ester groups are generally prone to hydrolysis under various conditions [38]. Therefore, the stability of two bioactive ester derivatives, RoyBz and the 12‐benzoyl derivative 7α‐acetoxy‐6β‐hydroxy‐12‐O‐benzoylroyleanone (Roy12Bz), [39, 40] was evaluated as representative examples. As previously demonstrated, Roy **1** showed poor solubility in PBS solutions, even with the use of co‐solvents. Similarly, RoyBz and Roy12Bz were insoluble in water and only moderately soluble in DMSO.

Stock solutions of each compound were prepared at 0.1 mM in DMSO/PBS. RoyBz formed a yellow solution with needle‐like crystals, while Roy12Bz resulted in a suspension. Both solutions were incubated at pH 7.4 and 37°C for 10 days. The resulting precipitates were collected, redissolved in methanol, and analyzed by HPLC. Each compound was identified by its retention time and UV spectrum. The chromatograms showed a single peak for each derivative, corresponding to the intact RoyBz and Roy12Bz compounds. These results confirm their stability under the tested conditions. Notably, Roy12Bz contains an ester group at C12, and RoyBz has esters at both C6 and C12, positions typically prone to hydrolysis. However, no ester cleavage was detected in either case. These findings support the feasibility of synthesizing new ester derivatives based on Roy **1** for therapeutic development.

## 3 Conclusions

This study explored the potential of novel theoretical royleanone derivatives as modulators of PKC‐δ, with a focus on applications in colon cancer therapy. Roy **1** served as a lead compound for structure‐based design, with modifications targeting the hydroxyl groups at positions C12 and C6. Docking studies revealed that aliphatic substitutions at C12 are favorable for PKC‐δ activation, while C6 can accommodate a broader range of functional groups. Among the tested derivatives, compound **16** showed the most promising binding profile. Overall, in silico screening identified several candidates worthy of further synthesis.

The interaction between Roy **1** and HSA was evaluated through steady‐state fluorimetric analysis. Results showed moderate binding affinity, with the main binding site located near the Trp‐214 residue in subdomain IIA (Sudlow site I). Furthermore, thermal denaturation studies revealed a significant increase in the melting temperature of the HSA/Roy complex compared to HSA alone, suggesting that Roy binding stabilizes the protein structure. These findings support the potential of HSA as a carrier for Roy **1** in systemic circulation.

Regarding compound stability, Roy **1** demonstrated full stability in aqueous medium at 0.1 mM, pH 7.4, and 37°C over a 40‐day period. Its stability appeared largely independent of concentration (0.1–0.4 mM). Similarly, the derivatives RoyBz and Roy12Bz remained intact, with no signs of ester cleavage under the same conditions. These findings reinforce the potential of Roy **1** as a robust lead molecule for the development of novel royleanone‐based therapeutics with enhanced biological activity.

## 4 Experimental

### 4.1 General

Essentially fatty acid‐free HSA, type A1887, was purchased from Sigma‐Aldrich (USA) and used without further purification. HSA concentration was determined using an extinction coefficient of 35 700 M^−1^ cm^−1^ at 280 nm. Roy **1** was dissolved in DMSO. All HSA‐related measurements were duplicated in 50 mM phosphate buffer, pH 7.4. Final concentrations of DMSO in HSA‐**1** mixtures did not exceed 1% (v/v).

### 4.2 Plant Materials

The plant material, *P. grandidentatus* Gürke, was cultivated in Parque Botânico da Tapada da Ajuda (Instituto Superior Agrário, Lisbon, Portugal) from cuttings obtained from the Kirstenbosch National Botanical Garden (Cape Town, South Africa). Voucher specimens (572/2008) were deposited in Herbarium João de Carvalho e Vasconcellos (ISA). The plant name has been checked using https://wfoplantlist.org/ [41].

### 4.3 Extraction and Isolation

The extraction and isolation process was adapted from Isca, V.M.S. et al. [23]. The leaves and stems of *P. grandidentatus* were air‐dried, ground into powder, and extracted with acetone in ultrasound equipment (Sonorex Super RK 510 H; Bandelin, Berlin, Germany). The isolation was performed by dry flash chromatography, using silica gel (Merck 9385) as the stationary phase and mixtures of Hex: AcOEt and AcOEt: MeOH as eluents. Roy**1** was obtained from recrystallization from Hex.

### 4.4 Molecular Docking of Theoretical Derivatives on PKC‐δ

The crystallographic structure of protein kinase delta CYS2 complexed with PA (1PTR.pdb from the protein data bank [21]) was used to perform the redocking of the known activator PA. The possible binding mode of RoyBz, reported as a PKC‐δ selective activator [18] was also assessed by molecular docking. FRED, Gold, and AutoDock programs were tested. FRED software [22] was selected to perform the docking studies. The binding pocket was defined according to Bessa et al. [18] Software Vida [42] was used to draw the structures' databases. Docking poses were visually inspected using Pymol software [43].

### 4.5 Molecular Dynamics Simulations

MD simulations were run with AMBER18, using the ff14SB force field for the protein, and the general Amber force field (GAFF) for the small molecule **16** [44, 45, 46]. The docking pose was solvated in a rectilinear box of TIP3P‐type explicit water molecules, buffering 15 Å from the macromolecular system, for a total of 6177 water molecules. The total charge of the system was neutralized by Cl^‐^ ions. The time step was 2 fs. According to a well‐established MD protocol [47, 48, 49, 50, 51], the initial system was first submitted to an energy minimization of the solvent only, while keeping the solute frozen, for 1000 steps with the steepest descent algorithm (SD), followed by 4000 steps with the conjugate gradient algorithm (CG). Then, the solvated solute was energy minimized for 1000 steps with the SD, followed by 9000 steps with the CG, before being heated to 300 K for 2 ns at constant volume using the Langevin thermostat. Density was equilibrated for 2 ns at constant pressure using the Berendsen barostat. A preliminary MD of 50 ns was run before the final production of MD trajectories lasting 500 ns at constant pressure. In all the steps above, no restraints were used. MD trajectory analysis was carried out with the CPPTRAJ software [52].

### 4.6 Fluorescence Spectroscopy Measurements

All fluorescence measurements were done on a FluoroMax‐4 spectrofluorometer (HORIBA Scientific, Japan) under temperature‐controlled conditions (Peltier control system), with the width of the excitation and emission slit both adjusted to 5 nm and with 1 cm path length cells. The fluorescence spectra were recorded under thermostated conditions (37°C). HSA concentration was kept constant (1 µM) while the Roy **1** concentration varied from 0–14 µM.

For intrinsic fluorescence experiments, the excitation wavelength was 280 nm, and emission spectra were recorded between 290 and 450 nm. The change in fluorescence emission intensity was measured within 1 min of adding each aliquot of ligand to the protein solution. The emission of Roy **1** solutions without HSA was subtracted to correct background fluorescence. Fluorescence intensities were corrected for inner filter effects according to the equation [53]:

$$F_{c}=F_{0}10^{\left(\mathrm{Aem}+\mathrm{Aex}\right)/2}$$

where Fc is corrected fluorescence, F0 is measured fluorescence, and Aex and Aem are the absorbances of Roy at excitation and peak emission wavelength, respectively.

The association (binding) constant for the HSA‐Roy complex was calculated using the equation [54]:

$$\log\frac{F_{0}-F}{F}=-n\log\frac{1}{\left[L\right]-\left[P\right]\;\frac{F_{0}-F}{F_{0}}}+n\log K_{a}$$

where [P] and [L] are the total concentrations of protein (HSA) and ligand (**1**), respectively, *K*_a_ is the binding constant, and n is the Hill coefficient (indication of the number of binding sites on the protein).

Synchronous fluorescence spectra of the HSA‐**1** complex, with emissions in the range of 290–400 nm, were obtained at two different scanning intervals: Δλ = 15 nm, tyrosine (Tyr) residues excitation, and Δλ = 60 nm, tryptophan (Trp) residues excitation, where Δλ = Δλ_em_—Δλ_ex_.

The thermal stability of HSA alone (1 µM) or in the presence of Roy **1** (10 µM) was studied in the temperature range from 25 to 85°C by measuring protein intrinsic fluorescence reduction. The rate of temperature increase was 2°C/min, with the equilibration time set to 1 min. The results were expressed as the change of the (fluorescence) ratio F330/F350 with the temperature and fitted with a sigmoidal function. The inflection point in the plot was taken as the melting point (T_m_) of the protein [55].

### 4.7 Antioxidant Capacity

The antioxidant capacity of Roy **1** was evaluated in six antioxidant assays: DPPH, ABTS, NO, TAOC, ORAC, and HORAC (Table 2) and compared with two standard antioxidants, Trolox (6‐hydroxy‐2,5,7,8‐tetramethylchroman‐2‐carboxylic acid) and Vitamin C (ascorbic acid). ABTS, DPPH, NO, and TAOC are spectrophotometric, while HORAC and ORAC are fluorometric assays. The percentage inhibition in ABTS, DPPH, and NO assays was calculated using the following equation:

$$\begin{array}{ccc} & & \mathrm{ABT}S^{+.}/\mathrm{DPPH}/\mathrm{NO}\;\mathrm{scavenging}\;\mathrm{effect}\;\left(\%\right)\\ & & =\left(\mathrm{Ai}\;-\;\mathrm{As}/A\;\mathrm{Control}\right)\;\times 100, \end{array}$$

where Ai is the initial concentration of the chromophore and As is the absorbance of the remaining concentration of chromophore in the presence of analyzed compounds. To compare the radical scavenging activity of Roy **1** with Trolox and Vitamin C in ABTS, DPPH, and NO assay, IC_50_ values were calculated from the graph of scavenging activity against the concentrations (from 50 µM to 2 mM) of the investigated compounds. The IC_50_ value is the sample concentration required for a 50% free radical scavenging inhibition activity.

In the TAOC test, antioxidant capacity was expressed as the EC_50_ value, which represents the concentration of the investigated compound that results in an absorbance of 0.500. An increase in absorbance is measured after mixing the solution of the studied compound with a mixture of (NH_4_)_6_Mo_7_O_24_, Na_3_PO_4_, and H_2_SO_4_.

The results of HORAC and ORAC assays are expressed as Trolox equivalents (TE). TE values are obtained from the equation: AUCS/AUCT, where AUCs = AUC(sample)—AUC(buffer blank) and AUCT = AUC(Trolox)—AUC(buffer blank) (AUC means the net area under the curve). In the case of both assays, reaction mixtures containing standards and fluorescein were used as substrates.

The principles of these assays (Table 4) may be found in the review book chapter written by Stengel et al. [56], while detailed experimental procedures are given in our recent papers [57, 58].

**TABLE 4:** Antioxidant assays used in this study.

Assay abbreviation	Full name of the antioxidant assay	Radical	Radical generation method/Mechanism of action
DPPH	(2,2‐diphenyl‐1‐picrylhydrazyl/DPPH) radical scavenging assay	DPPH^●^	—
ABTS	(2,2′‐azino‐bis[3‐ethylbenzothiazoline‐6‐sulfonic acid]/ABTS) radical scavenging assay	ABTS^+●^	The reaction of ABTS with K_2_S_2_O_8_ in water
NO	Nitric oxide scavenging	_●_NO	Incubation of Na_2_[Fe(CN)_6_] solution in light
HORAC	Hydroxyl radical antioxidant capacity	^●^OH	H_2_O_2_/CoF_2_/picolinic acid
ORAC	Oxygen radical absorbance capacity	^●^OOH	Thermal homolysis of 2,2’‐azobis (2‐amidino‐propane) dihydrochloride (AAPH)
TAOC	Total antioxidant capacity assay	−	The reduction of Mo(VI) to Mo(V), with the subsequent formation of a stable blue‐green phosphate Mo(V)complex at acidic pH.

### 4.8 Evaluation of Roy Stability in Aqueous Medium

Stock solutions of Roy **1** (0.4 mM) were prepared in PBS (pH 7.4) containing 10% of DMSO (9 mL PBS to 1 mL DMSO), adapted from Mak et al., 2017 [37]. Solutions were incubated at 37°C for 10 days. One of the stock solutions (PBS/DMSO) was kept under constant stirring during the assay. Stock solutions of compound **1** (0.1 mM and 0.4 mM) were prepared in PBS buffer (pH 7.4), containing 17% acetonitrile (ACN) (20 mL PBS to 4 mL ACN) [59]. Solutions were incubated at 37°C for 40 and 10 days, respectively. At different times, several aliquots of the reaction mixtures were taken and analyzed by HPLC‐DAD at 270 nm wavelength. The compounds RoyBz and the 12‐benzoyl derivative Roy12Bz were previously synthesized [39, 40]. Stock solutions of RoyBz and Roy12Bz (0.1 mM) were also tested in PBS (pH 7.4) and 10% of DMSO at 37°C for 10 days. The quantification was carried out in an Agilent Technologies 1200 Infinity Series system with a diode array detector (DAD; Agilent, Santa Clara, CA, USA) equipped with an Eclipse XDB‐C18 (250 x 4.0 mm i.d., 5 µm) column, from Merck and ChemStation Software (Hewlett‐Packard, Palo Alto, CA, USA). Each sample was analyzed (after 20 µL injection), and a gradient elution mixture composed of solution A (methanol), solution B (acetonitrile), and solution C (0.3% trifluoroacetic acid in water) was used as follows: 0 min, 15% A, 5% B, and 80% C; 2 min, 70% A, 30% B, and 0% C; 10 min, 70% A, 30% B, and 0% C; and 15 min, 15% A, 5% B, and 80% C. The flow rate was set at 1 mL.min^−1^. The column was maintained at 29°C. The analysis time was 15 min, including the stabilization of the RP‐18 column. A calibration curve with acceptable validation parameters was constructed. All samples were filtered before HPLC analysis. Assay conducted in triplicate.

## Author Contributions

**Patricia Rijo** and **Mattia Mori** conceived the experiments; **Vera M. S. Isca** and **Milan Nikolić** conducted the experiments; and **Vera M. S. Isca** and **Nenad Filipović** analyzed the results. All authors reviewed the manuscript.

## Conflicts of Interest

The authors declare no conflict of interest.

## Data Availability

The data supporting the findings of this study are available from the corresponding author upon reasonable request.
